# Arsenic trioxide demonstrates efficacy in a mouse model of preclinical systemic sclerosis

**DOI:** 10.1186/s13075-023-03143-2

**Published:** 2023-09-12

**Authors:** Anne Cauvet, Arthur Decellas, Christophe Guignabert, Dominique Rongvaux-Gaïda, Raphaël Thuillet, Mina Ottaviani, Ly Tu, François Rieger, Jérôme Avouac, Yannick Allanore

**Affiliations:** 1 Université de Paris, Institut Cochin, INSERM U1016 CNRS UMR8104, Paris, 75014 France; 2 UMR_S 999 “Pulmonary Hypertension: Pathophysiology and Novel Therapies”, INSERM, Hôpital Marie Lannelongue, Le Plessis-Robinson, France; 3 Faculté de Médecine, Pulmonary Hypertension: Pathophysiology and Novel Therapies, Université Paris-Saclay, Le Kremlin-Bicêtre, France; 4 MEDSENIC SAS, Strasbourg, France; 5 Rheumatology Department, Université de Paris, Cochin Hospital, Paris, France

**Keywords:** Systemic sclerosis, Arsenic trioxide, Pulmonary fibrosis, Pulmonary hypertension

## Abstract

**Background:**

Uncontrolled T-cell activation plays a key role in systemic sclerosis (SSc). Arsenic trioxide (ATO) has immunological effects and has demonstrated potential in preclinical SSc models. In this study, we assessed the efficacy of ATO in Fra2 transgenic (Fra2^TG^) mice, which develop severe vascular remodeling of pulmonary arterioles and nonspecific interstitial pneumonia-like lung disease, closely resembling human SSc-associated pulmonary hypertension, therefore partially resembling to the SSc human disease.

**Methods:**

The efficacy of ATO in Fra2^TG^ mice was evaluated through histological scoring and determination of cell infiltration. Fibrotic changes in the lungs were assessed by measuring collagen content biochemically, using second harmonic generation to measure fibrillar collagen, and imaging via computed tomography. Cardiovascular effects were determined by measuring right ventricular systolic pressure and vessel remodeling. The mechanism of action of ATO was then investigated by analyzing lung cell infiltrates using flow cytometry and bulk RNA with sequencing techniques.

**Results:**

After ATO treatment, the Ashcroft histological score was substantially decreased by 33% in ATO-treated mice compared to control mice. Other investigations of fibrotic markers showed a trend of reduction in various measurements of fibrosis, but the differences did not reach significance. Further cardiovascular investigations revealed convergent findings supporting a beneficial effect of ATO, with reduced right ventricular systolic pressure and medial wall thickness, and a significant decrease in the number of muscularized distal pulmonary arteries in ATO-treated Fra2^TG^ mice compared to untreated Fra2^TG^ mice. Additionally, inflammatory cell infiltration was also markedly reduced in lesioned lungs. A reduction in the frequency of CD4 + and T effector memory cells, and an increase in the percentage of CD4 + T naive cells in the lungs of ATO-treated Fra-2^TG^ mice, was observed when compared to PBS group Fra-2^Tg^ mice. RNA-seq analysis of ATO-treated mouse lungs revealed a downregulation of biological pathways associated with immune activity and inflammation, such as T-cell activation, regulation of leucocyte activation, leucocyte cell–cell adhesion, and regulation of lymphocyte activation.

**Conclusions:**

Our results suggest the clinical relevance of ATO treatment in SSc. Using the Fra2^TG^ mouse model, we observed significant lung histological changes, a trend towards a decrease in various fibrotic makers, and a strong reduction in vascular remodeling. The mechanism of action of ATO appears to involve a marked counteraction of the immune activation characteristic of SSc, particularly T-cell involvement. These findings pave the way for further studies in SSc.

**Supplementary Information:**

The online version contains supplementary material available at 10.1186/s13075-023-03143-2.

## Introduction

Systemic sclerosis (SSc) is a rare and incurable connective tissue disease characterized by microvascular damage, extensive immune abnormalities, and fibrosis of skin and internal organs [1]. SSc is recognized as the most life-threatening rheumatic disease, with pulmonary complications such as interstitial lung disease (ILD) and pulmonary hypertension (PH) being the primary causes of mortality [2].

Although the pathogenesis of SSc is complex and not yet fully understood [3], research has shown that immune dysfunction is one of the key components of the disease [4, 5].

Compelling evidence suggests that various cells and soluble mediators belonging to both innate and adaptive systems exhibit abnormalities that are significantly associated with distinct SSc phenotypes, potentially contributing to disease development and progression. The altered cell behavior sustaining tissue damage in SSc seems to be influenced by radical oxygen species and the activation of the invariant receptors sensing danger. Fibrosis in SSc is thought to be due to an inflammatory response to chronic tissue injury, with the enhanced deposition of extra-cellular matrix not fully compensated for by its natural resorption. Fibrosis in SSc can be seen as a process resembling wound healing, where the resolution phase is ineffective or even absent, with indications linking immune disturbances to mesenchymal cell overactivation [6].

Over the past few years, several inducible mouse models of SSc have been developed, such as models of bleomycin-induced or hypochlorous acid-induced fibrosis and the sclerodermatous graft-versus-host disease (GVHD) model. However, none of these models fully encompass all the features of SSc. Specifically, they do not exhibit the specific lung tissue and lung vascular damage observed in humans [7]. Recently, a new Fra2 transgenic mouse (Fra2^TG^) model has been established. These mice overexpress the AP-1 transcription factor Fra2, encoded by *Fosl2* [8], leading to the development of spontaneous systemic inflammation and fibrosis, primarily in the lung and skin [8]. In the lungs, overexpression of Fra2 results in an age-dependent increase in perivascular and peri-bronchial inflammation, severe fibrosis, and premature death. Before the onset of pulmonary fibrosis, these mice exhibit vascular remodeling with collagen deposition in the vessel walls and increased vessel muscularization, which leads to pulmonary hypertension [9]. This genetic model is of great interest as it mirrors the sequential pathological events observed in humans [7]. Several therapeutics that target T-cell costimulatory pathways, including OX40L [10], CTLA-4 [11], and a dual ICOS/CD28 antagonist [12], have been shown to reduce both the lung abnormalities and pathologic remodeling in Fra2^TG^ mice.

Arsenic trioxide (ATO; As_2_O_3_) has been approved for the treatment of acute promyelocytic leukemia (APL) [13]. Its effects on cells are complex and vary depending on cell types. ATO has been found to have a direct pro-apoptotic effect on leukemic cells and decrease the amount and function of Treg cells in the treatment of APL. In a murine islet allotransplantation model, ATO prolonged islet allograft survival by increasing the proportion of Foxp3 + Treg cells and inhibiting proliferation of T lymphocytes and decreasing B lymphocytes [14]. In models of pre-clinical SSc, ATO has demonstrated its ability to reduce clinical symptoms and mainly skin fibrotic changes in sclerodermatous GVHD and in HOCl-induced SSc. These beneficial effects were mediated through the depletion of glutathione and the overproduction of H_2_O_2_ that killed activated CD4( +) T cells and plasmacytoid dendritic cells (pDCs) [15]. ATO also reduced vascular damage, TH2 cytokines, and autoantibodies [16]; taken together, these data support a potential benefit of ATO as a treatment of SSc.

Here, we aimed to investigate the effects of ATO on Fra2^TG^ mice to gain insights into the lung manifestations related to SSc.

## Materials and methods

### Animals

Female Fra-2^TG^ mice (*B6.Cg-Tg(H2-KFosl2, EGFP)13Wag*) were housed in a specific and opportunistic pathogen-free facility (C75-14–02) at the Cochin Institute, Paris, France. Animal experiments were conducted in accordance with the Guide for the Care and Use of Laboratory Animals adopted by the French National Institute of Health and Medical Research (Inserm) and were approved by the Ethics Committee of Université Paris Cité.

### *Fra-2*^*TG*^* mice and ATO treatment*

Transgenic mice that express the *fra-2* gene, under the control of the ubiquitous major histocompatibility complex class I antigen H-2 Kb promoter, exhibit systemic fibrosis, microangiopathy, and pulmonary hypertension (PH) [17].

Two groups of female Fra-2^TG^ mice were treated with intraperitoneal injections of either a phosphate-buffered saline (PBS) control solution (PBS group; *n* = 11), or arsenic trioxide (ATO) at 5 μg/g (ATO group; *n* = 11), beginning at 12 weeks of age. The injections were administered for 5 consecutive days per week for a total of 6 weeks. At 18 weeks of age, the mice were euthanized by exsanguination after measurement of the right ventricular systolic pressure (RVSP). The ATO used (Arscimed, clinical grade batch, stock solution at 1 mg/ml) was obtained from MEDSENIC (Strasbourg, France).

### *Clinical follow-up of Fra-2*^*TG*^* mice*

Fra2^TG^ mice develop a disease phenotype that requires careful clinical monitoring on a daily basis. Both experimental groups were weighed and individually evaluated for physical appearance and general behavior on a weekly basis. A clinical score ranging from 0 to 10 was assigned to each mouse based on the extent of body weight loss (scored from 0 to 3, with 0 indicating no weight loss, 1 indicating weight loss of less than 10%, 2 indicating weight loss between 10 and 20%, and 3 indicating weight loss of over 20%) as well as the presence or absence of symptoms such as blepharitis, alopecia, skin sclerosis, hepatomegaly, ruffled fur, hunched posture, and lethargy.

### Histopathologic assessment of fibrosing alveolitis

The left lung was excised from sacrificed Fra2^TG^ mice and embedded in paraffin. Lung Sects. 4-μm-thick were stained with hematoxylin–eosin. The severity of fibrosing alveolitis was semi-quantitatively assessed, using the method described by Ashcroft et al., by two examiners blinded to the genotype and treatment [18]; the left upper lobe was examined, and fibrosis was graded on a scale of 0 to 8. The grading criteria were as follows: grade 0 = normal lung; grade 1 = minimal fibrous thickening of alveolar walls; grade 3 = moderate thickening of walls without obvious damage; grade 5 = increased fibrosis with definite damage and formation of fibrous bands; grade 7 = severe distortion of structure and large fibrous areas; and grade 8 = total fibrous obliteration. Grades 2, 4, and 6 represent intermediate stages between these key criteria. All images were scanned using a Lamina Multilabel Slide Scanner (Perkin Elmer, Waltham, MA) and analyzed using CaseViewer software (version 2.4).

### Immunohistochemistry for anti-CD3, anti-CD68 and anti-Ly6G

Cell infiltration was identified in paraffin-embedded sections of the lung from the ATO and PBS groups. After antigen retrieval, blocking and tissue permeabilization with PBS + 0.25% Triton X-100, lung sections were incubated overnight at 4 °C with the following primary antibodies diluted in PBS + 0.5% BSA: rabbit anti-CD3 (Abcam, clone, dilution 1/100), rat anti-Ly6G (BD Biosciences, Clone 1A8, dilution 1/500), rabbit anti-CD68 (Abcam, dilution 1/100). Polyclonal goat anti-rabbit (1/200) or goat anti-rat antibodies (Invitrogen, dilution 1/500) labeled with horseradish peroxidase (HRP) were used as secondary antibodies for 1 h at room temperature. Immunoreaction products were revealed using diaminobenzidine (DAB) solution (Liquid DAB + Substrate Chromogen System, Dako, Denmark). The Lamina Multilabel Slide Scanner was used to analyze the immunostaining, and slide staining analysis was performed with CaseViewer software (version 2.4).

### Collagen measurements in Fra-2 transgenic mice

Collagen content was measured by hydroxyproline assay, as previously described [19]. Lung biopsies (from the right lobe for each mouse) were hydrolyzed and titrated to pH 7. This solution was combined with chloramine T and p-dimethylaminobenzaldehyde in perchloric acid and read at 557 nm with a spectrophotometer (Molecular Devices, Sunnyvale, CA).

### Nonlinear microscopy and second harmony generation (SHG) processing

A 2-photon Leica SP8 DIVE FLIM (Leica Microsystems GmbH, Wetzlar, Germany) was used for lung tissue imaging. Two lasers at 1040- and 880-nm wavelength were used to generate SHG and two-photon-excited fluorescence (TPEF) signals, collected by a Leica Microsystems HCX IRAPO 25 × /0.95 W objective and two external detectors. Microscopy was performed on 16-μm-thick blank blades of sliced lungs. Five samples of each slice were taken. The SHG score was established by comparing the area occupied by the collagen relative to the sample surface. Image processing and analysis (thresholding and SHG scoring) were performed using an ImageJ homemade routine, as previously described [20].

### Assessment of fibrosing alveolitis by chest micro-computed tomography (micro-CT)

Fibrosing alveolitis of mice was evaluated using micro-CT 2 days before sacrifice, as previously reported [20]. CT images were obtained with a Perkin Elmer Quantum FX system (Caliper Life Sciences, GmbH, Mainz, Germany). The animals were placed in the supine position on the CT table and sedated with 3–4% isoflurane anesthesia under 0.5–1.5 L/min for induction by a nose cone. Anesthesia was maintained with 2.5–3% isoflurane under 400–800 mL/min during the acquisition. During image acquisition thoracic breathing movements were recorded, detecting the up- and downward movements of the thorax. Images were acquired throughout the spontaneous respiratory cycle. Only images acquired during expiration were analyzed. Images were acquired with the following parameters: 90 kV X-ray source voltage, 160 μA current. Total scanning time was approximately 4.5 min per mouse. Means of lung density of both groups were achieved by evaluation of all CT scans acquired from the apices to the bases of the lungs. Furthermore, the volume of functional lung parenchyma corresponding to functional residual capacity (FRC) was manually drawn, by excluding the fibrosis area and vessels. Percentages of FRC on total lung volumes were calculated. The CT expert (FT) was blinded to the background of the mice, to the treatment, and to the results of the histological assessment.

### Quantitative RT-PCR

Total lung RNA was extracted from lung biopsies with TRIzol reagent (Invitrogen, Waltham, MA); 1 μg of RNA was treated with 1 unit of DNase and reversed transcribed into cDNA (Invitrogen) according to the manufacturer’s instructions. Quantitative PCR amplification was performed with specific FAM dye-labeled TaqMan probes for Col1a1, Col1a2, Fn1, Actb (Invitrogen) and the detection system 7300 real Time PCR system (Applied Biosystem, Waltham, MA. Results were expressed as the ratio of the target gene, normalized to Actb.

### Hemodynamic measurements and assessment of vessel remodeling

RVSP and heart rate were measured in unventilated mice under isoflurane anesthesia (1.5–2.5%, 2 L O_2_/min) using a closed chest technique. A catheter (1.4-F catheter; Millar Instruments Inc., Houston, TX) was introduced into the jugular vein and directed to the right ventricle. After completing all hemodynamic assessments, blood was collected by direct cardiac puncture, and the mice were sacrificed by exsanguination. The heart and lungs were then removed and flushed with 5 mL buffered saline at 37 °C, and then the left lung was prepared for morphometric analyses, and the right lung was quickly harvested, snap-frozen in liquid nitrogen, and stored at − 80 °C. Morphometric analyses were performed on paraffin-embedded lung sections stained with hematoxylin and eosin and alpha smooth muscle actin (α-SMA). α-SMA was detected by incubating with monoclonal anti-α-SMA antibody (clone 1A4; Dako Glostrup, Denmark) at a dilution of 1:100 overnight at 4 °C. A Vectastain ABC kit was used according to the manufacturer’s instructions (Vector Laboratories, Burlingame, CA), and slides were then counterstained with hematoxylin (Sigma-Aldrich). The percentage of wall thickness ([2 × medial wall thickness/external diameter] × 100) and of muscularized vessels was determined as previously described [21]. All morphometric analyses were performed by two examiners (MO and CG) blinded to genotype and treatment conditions.

### Lung cell isolation for flow cytometry staining

Flow cytometry staining was performed on 11 mice from each group. The lung biopsies were incubated in PBS 10% FBS + Collagenase II (1 mg/mL), and DNase I (0.1 mg/mL), both from StemCell Technologies, Vancouver, Canada) for 1 h at 37 °C. The digested tissues were filtered through a 70-μm strainer (BD Biosciences). Red blood cells were lysed with ACK Lysing Buffer (Thermo Fisher). A Percoll (Sigma-Aldrich, St. Louis, MO) density gradient was generated by resuspending cells in a 40% Percoll solution and adding an 80% Percoll solution below the 40% solution. The cells were collected in the interface, washed, and resuspended in 2% FCS in PBS for flow cytometry cell surface staining. Firstly, the cells were incubated with Zombie Dye UV (Biolegend, San Diego, CA) for 15 min at room temperature. Fc receptors were blocked with the TruStain FcXTM (anti-mouse CD16/32) Antibody (Clone 93, Biolegend) for 5 min on ice. To investigate the effect of ATO in T-cell activation, we used antibodies directed against CD4 (Clone GK1.5) and CD8 (Clone 53–6.7). Activation of CD4 + and CD8 + T cells was assessed based on the expression of the early activation marker CD69 (anti-CD69-BV650; Clone H1.2F3) and the T-cell exhaustion marker PD-1 (anti-PD-1-PE/Dazzle 594 (Clone 29F.1A12). The proportions of effector memory T cells (TEM), central memory cells (TCM), and naive T cells (T Naive) were assessed based on their differential expression of CD62L (anti-CD62L-APC/Cy7 antibody; Clone MEL-14) and CD44 (anti-CD44- BV605; Clone IM7). Antibodies were purchased from Biolegend, except anti-CD8 (Clone 53–6.7) purchased from BD Biosciences. Stained cells were fixed in PBS 2% PFA. Data acquisition was performed on a BD LSR Fortessa Cytometer, and data were analyzed with FlowJo Software (version 10.7.2).

### Transcriptomic approach

We analyzed mRNA from 11 lung biopsies from PBS-treated Fra2^TG^ mice and 11 from lung biopsies from ATO-treated Fra2^TG^ mice. Preparation of RNA library and transcriptome sequencing was conducted by Novogene (Cambridge, UK). Prior to differential gene expression analysis, for each sequenced library, the read counts were adjusted using the edgeR program package through one scaling normalized factor. Differential expression analysis of two conditions was performed using the edgeR R package (3.22.5). The *p*-values were adjusted using the Benjamini and Hochberg method. Corrected *p*-value of 0.05 and absolute foldchange of 2 were set as the threshold for significantly differential expression. Enrichment analysis of differentially expressed genes was implemented using the clusterProfiler R package, in which gene length bias was corrected. Gene Ontology (GO) terms with corrected *p*-value < 0.05 were considered significantly enriched by differentially expressed genes.

### Statistical analyses

The data are expressed as the median [IQR]. Mann–Whitney tests were used to assess the statistical significance of differences between the two groups. Differences were considered significant for *p*-values < 0.05. Analyses were performed using PRISM software (version 8.3.0) (GraphPad, la Jolla).

## Results

### *Safety assessment of ATO treatment in Fra2*^*TG*^* mice*

At 5 μg/g during 6 weeks, the ATO treatment was well-tolerated, and the Fra2^TG^ mice in both the PBS and ATO groups maintained their mean body weight throughout the experiment (Fig. S1A). From the 4th week of treatment, a slight trend towards a decreased clinical score was observed in the ATO-treated Fra2^TG^ mice as compared to the PBS group (median score [IQR]: 1 [1–2.75] vs. 2 [1–4]; *p* = 0.071) (Fig. S1B).

### ATO treatment attenuates inflammatory cell infiltration in lesional lungs

**Fig. 1:**
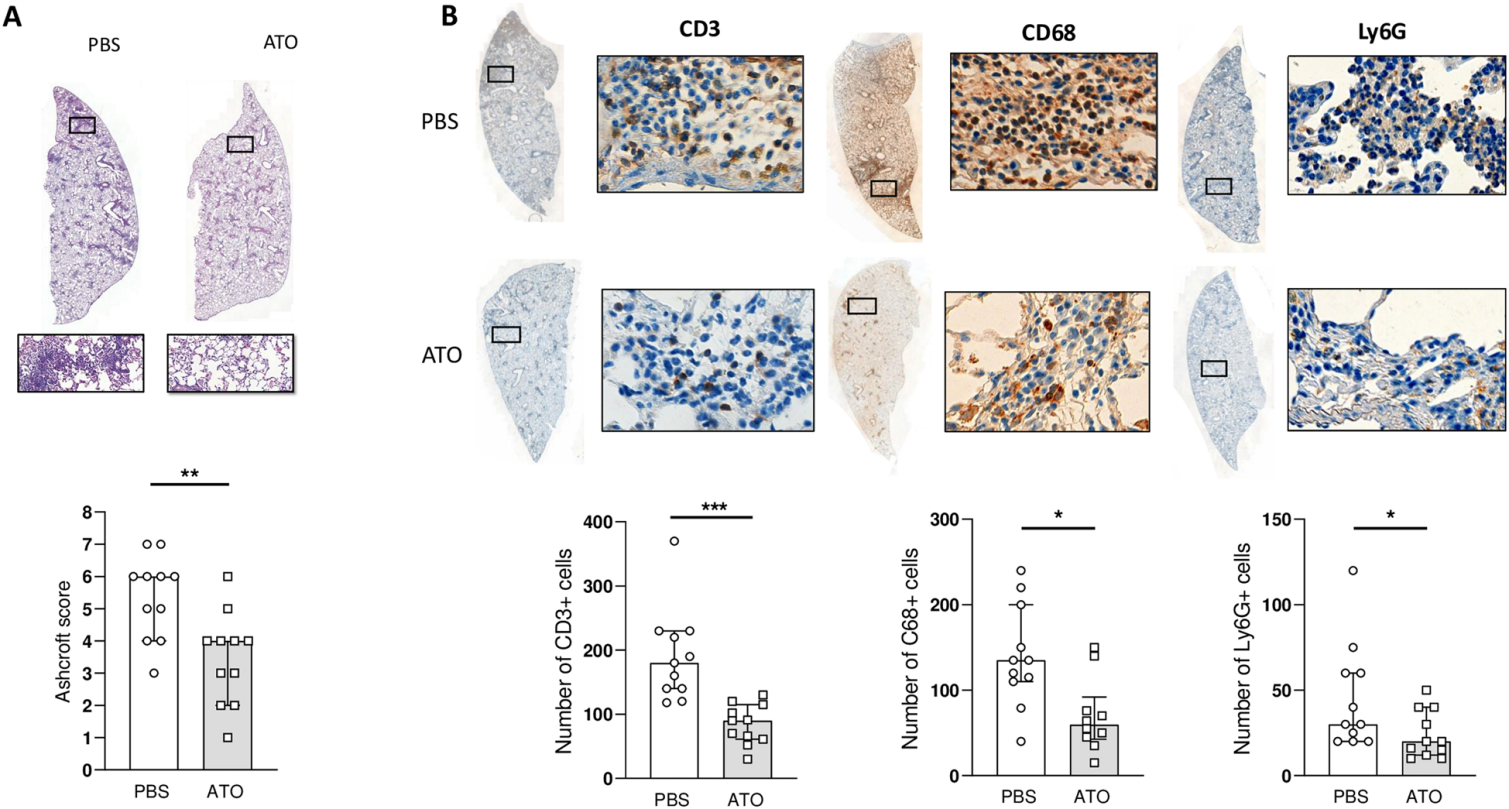
ATO alleviates inflammatory cell infiltration in the lung of Fra-2 transgenic mice. **A** Top: representative lung sections stained by hematoxylin and eosin. (magnification × 8). Bottom: histological lung fibrosis score (Ashcroft scale). **B** Top: representative IHC staining of T cells (CD3 +), macrophages (CD68 +), and neutrophils (Ly6G +) on 4-μm-thick lung section counterstained with hematoxylin (magnification: left × 10; right × 100). Bottom: Count number of CD3, CD68, and LY6G cells. Fra-2.^TG^ mice were divided into 2 groups: PBS group (*n* = 11) and ATO group (*n* = 11). Values are represented by plot; bars represent the median with interquartile range. Statistics are from Mann-Whitney *U* test * = *p* < 0.05; ** = *p* < 0.01; *** = *p* < 0.001

Lung sections from control Fra2^TG^ mice showed extensive perivascular and peribronchiolar inflammatory infiltrate, as well as collagen deposition. ATO-treated Fra2^TG^ mice exhibited a significant 33% decrease in the histological Ashcroft score of fibrosis compared to control-treated Fra2^TG^ mice (median score [IQR]: 4 [2–4] vs. 6 [4–6]; *p* = 0.0068) (Fig. 1A). Within the inflammatory infiltrates, T cell counts were markedly reduced by 57%, (90 [61–115] vs. 180 cells [140–230]; *p* = 0.001) in mice treated with ATO as compared to those receiving PBS (Fig. 1B). Additionally, ATO treatment led to a 56% reduction in the number of macrophages (59.5 [42.5–92] vs. 135 cells [110–200]; *p* = 0.01), and a 33% reduction in the number of neutrophils (20 [12–40] vs. 30 cells [20–60]; *p* = 0.031), as compared to the PBS group.

### Assessment of effect of ATO treatment on multiple outcome measures related to lung fibrosis

**Fig. 2:**
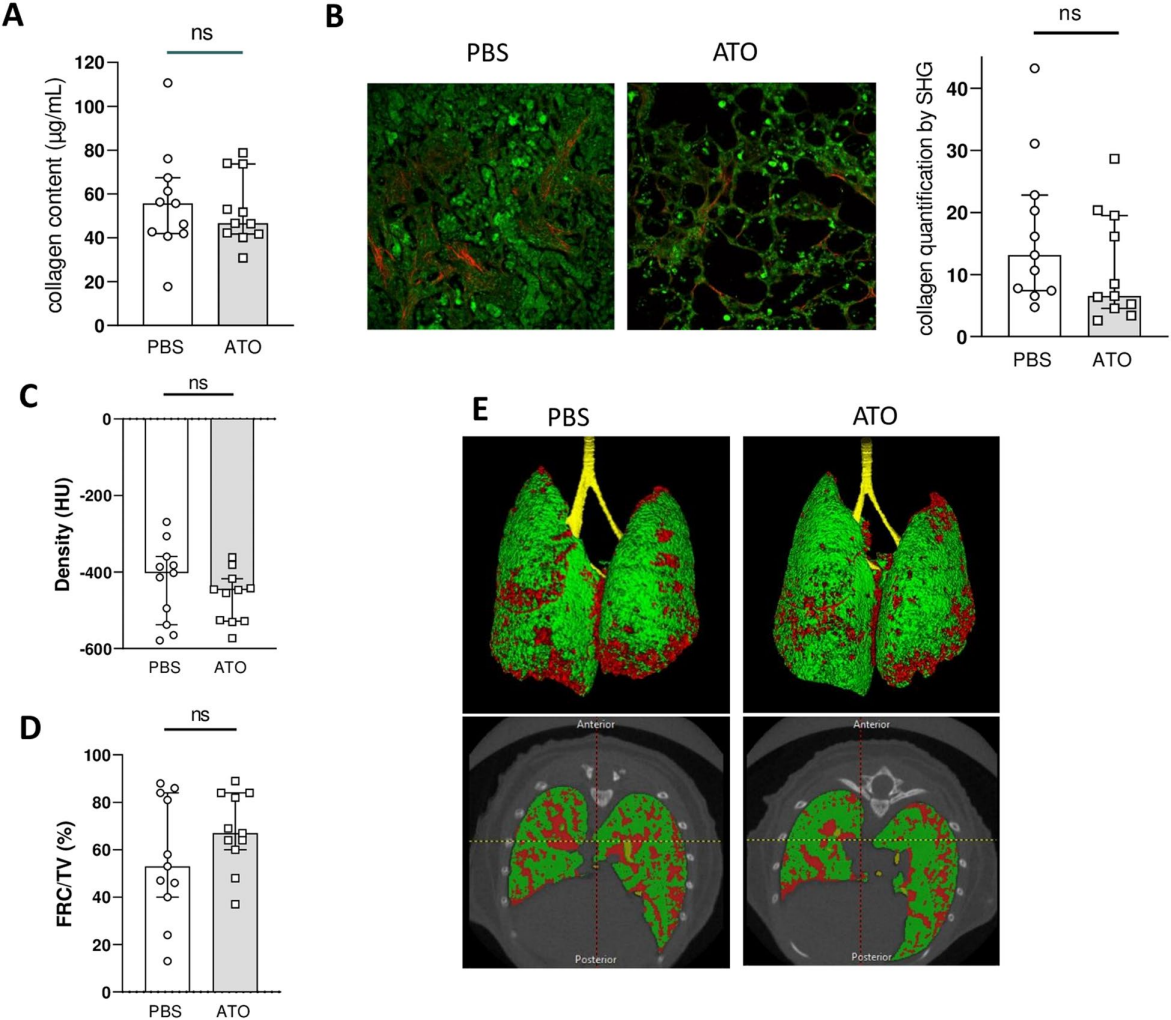
Evaluation of ATO effects on various lung fibrosis outcome measures. **A** Content of collagen in a lung fragment assessed by hydroxyproline assay. **B** Left: representative SHG images of 16-μm-thick lung sections (magnification × 25); Right: scoring of fibrillar collagen in lung section assessed by SHG. **C** Lung density at microcomputed tomography (micro-CT). **D** Residual lung volume, expressed as the percentage of functional residual capacity. **E** Representative micro-CT images. Fra-2^TG^ mice were divided into 2 groups: PBS-treated mice (*n* = 11) and ATO-treated mice (*n* = 11). Values are represented by plot; bars represent the median with interquartile range. Statistics are from Mann-Whitney *U* test. ns = not significant

We next investigated the efficacy of ATO in preventing lung fibrosis. Our analysis showed that mice receiving ATO treatment had a mild reduction in hydroxyproline levels compared to those injected with PBS, but this difference was not statistically significant in PBS groups (46.5 [41.7–73.65] μg/mL in ATO vs. 55.12 [41.91–67.35] μg/mL; *p* = 0.7) (Fig. 2A).

SHG microscopy revealed perivascular and interstitial fibrosis in the control Fra2^TG^ mice, indicating fibrosing alveolitis (Fig. 2B). Although ATO treatment led to a 50% reduction in fibrillar collagen deposits compared to PBS treatment, this difference did not reach statistical significance (6.53 [4.5–19.52] vs. 13.13 [7.41–22.79]; *p* = 0.17) (Fig. 2B).

We also analyzed chest tomography, which provided quantitative data on lung parenchymal changes and breathing capacities. Both lung density and functional residual capacity (FRC) were reduced in Fra2^TG^ ATO-treated mice compared to PBS-treated mice, although these differences did not reach statistical significance. The median lung density was − 447 HU [− 528, − 417] in the ATO group compared to − 402 HU [− 538, − 359.5] in the control group (*p* = 0.36). The median FRC value was 67% [60–84] in ATO-treated mice and 53% [40–84] in the PBS group (*p* = 0.26) (Fig. 2C–E).

The molecular biology analysis revealed similar results, with a trend for collagen reduction in the lungs (Col1a1, Col1a2) of mice treated with ATO compared to those treated with PBS, but the difference was not statistically significant (Fig. S2).

### *ATO treatment reduces vascular remodeling and pulmonary hypertension in Fra-2*^*TG*^* mice*

**Fig. 3:**
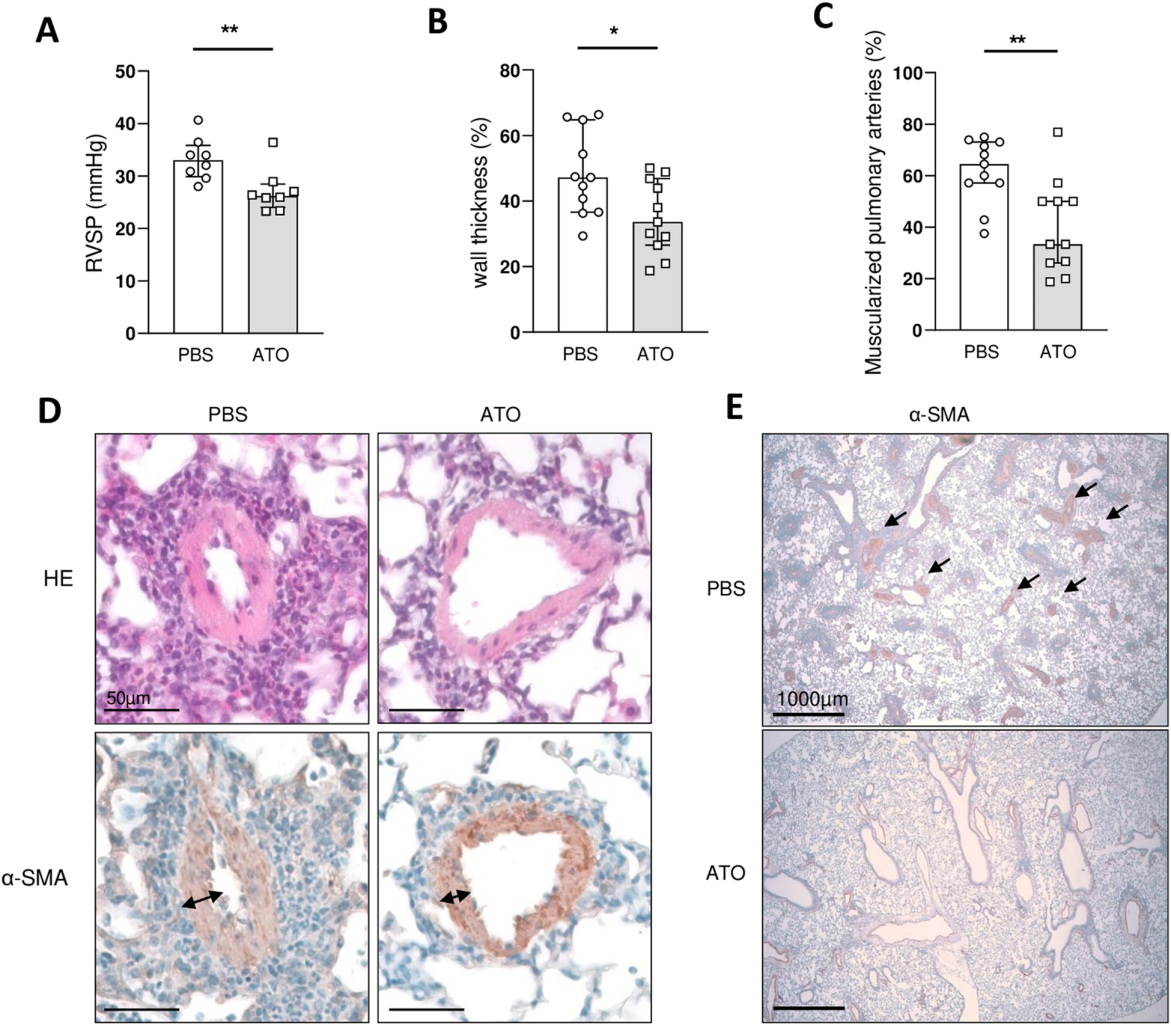
ATO alleviates pulmonary hypertension in Fra-2 transgenic mice. **A** Right ventricular systolic pressure (RVSP). **B** Percentage of medial wall thickness. **C** Percentage of distal artery muscularization. **D** Top: representative images of hematoxylin and eosin staining. Bottom: representative images of a smooth muscle actin (αSMA). **E** Representative images of a smooth muscle actin (αSMA). Fra-2^TG^ mice were divided into 2 groups: PBS group (*n* = 11) and ATO group (*n* = 11). Values are represented by plot; bars represent the median with interquartile range. Statistics are from Mann-Whitney *U* test. * = *p* < 0.05; ** = *p* < 0.01

We then investigated the cardiovascular changes in Fra2^TG^ mice. We compared mice treated with ATO to those that received PBS injections. The results showed that ATO treatment significantly reduced pulmonary hypertension, with a 21% decrease in RVSP in ATO-treated Fra2^TG^ mice compared to PBS mice (26.18 mmHg [24.00–28.44] vs. 32.99 mmHg [29.89–35.82]; *p* = 0.007) (Fig. 3A). We also examined vascular remodeling and found that medial wall thickness was significantly decreased by 29% in mice receiving ATO injection compared to those receiving PBS (33.63 [26.53–46.86] vs. 47.19% [36.61–64.8]; *p* = 0.03) (Fig. 3B, D). Furthermore, the number of muscularized distal pulmonary arteries was reduced by 48% in ATO-treated mice compared to those receiving PBS (33.33 [26–50] vs. 64.52 [57.14–73.08]; *p* = 0.0062) (Fig. 3C, E).

### *ATO treatment reduces CD4* + *TEM frequency and increases CD4* + *TCM and T naive cells in Fra-2*^*TG*^* mouse lungs*

**Fig. 4:**
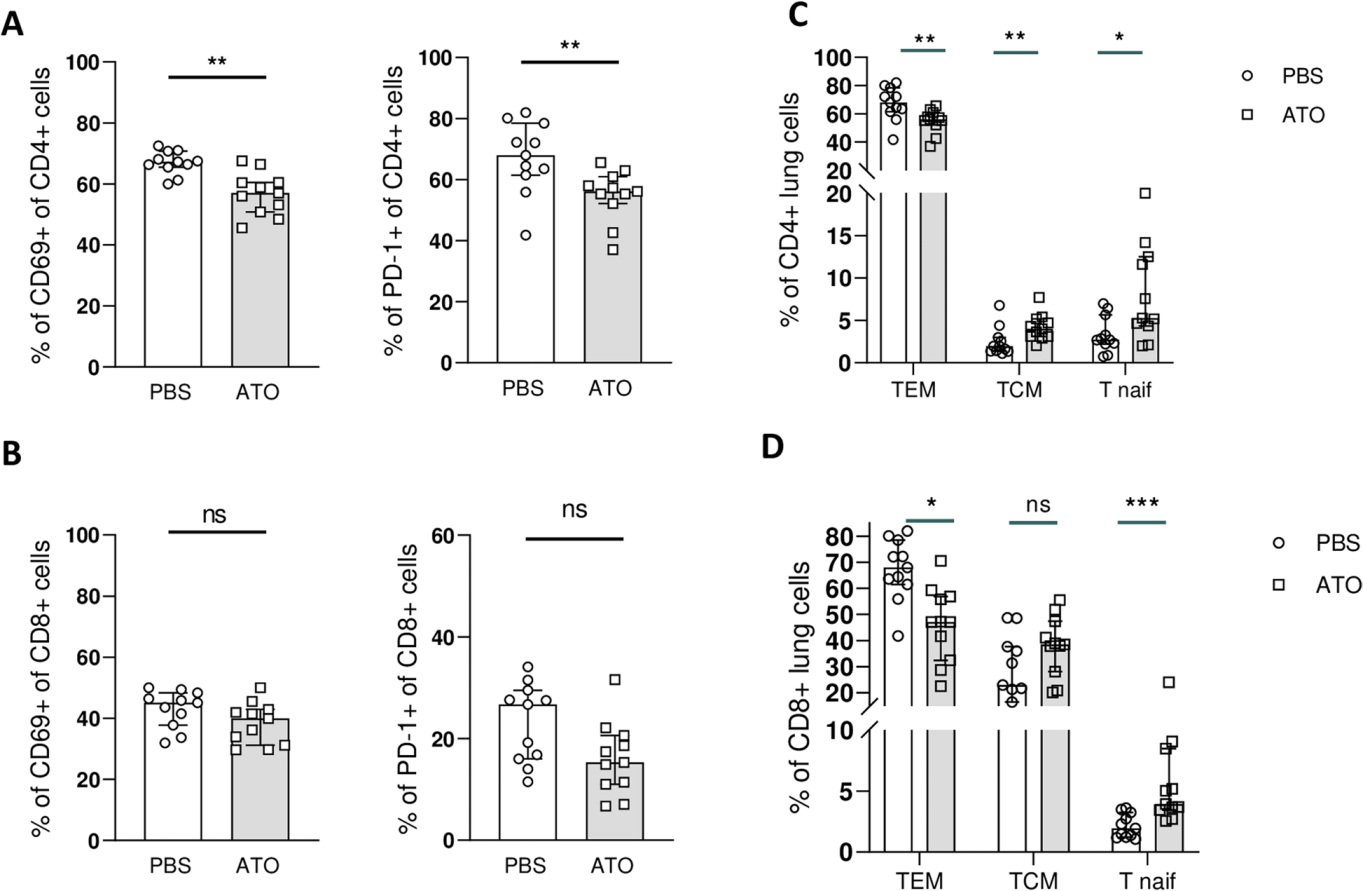
ATO decreased T-cell activation in Fra-2 Tg lung cells. **A** Frequencies of CD69 + and PD1 + cells within CD4 + subset in lungs. **B** Frequencies of CD69 + and PD1 + cells within CD8 + subsets in lungs. **C** Frequencies of CD4 + effector memory T cells (TEM CD62L-CD44 +), central memory T cells (TCM: CD62L + CD44 +), and percentage of naive T cells (CD62L + CD44-) in lungs of both treated groups. **D** Frequencies of CD8 + effector memory T cells (TEM CD62L-CD44 +), central memory T cells (TCM: CD62L + CD44 +), and percentage of naive T cells (CD62L + CD44-) in lungs of both treated groups. Flow cytometry analysis was performed on 11 lungs from the PBS group and 11 lungs from ATO-treated Fra-2^TG^ mice. Values are represented by plot; bars represent the median with interquartile range. Statistics are from Mann-Whitney *U* test. * = *p* < 0.05; ** = *p* < 0.01; *** = *p* < 0.001; ns = not significant

Treatment with ATO resulted in a significant decrease (15%) in the fraction of CD69-expressing cells within the CD4 + subset in the ATO-treated mice lung (57% [50.8–60.5] vs. 67.2% [65.5–70.7]; *p* = 0.001) (Fig. 4A). However, no changes in the frequency of CD69-expressing cells were detected within the CD8 + T cell subset in lungs (Fig. 4B). ATO treatment also led to a decrease in PD-1-expressing cells within both the CD4 + (56.2% [52.2–61] vs. 68% [61.5–78.5]; *p* = 0.006) and CD8 + T cell subsets (15.3% [11–20.6] vs. 26.7% [16–29.8]; *p* = 0.06] in the ATO-treated mice lung (Fig. 4A, B). ATO treatment also induced a significant decrease (17%) in the frequency of CD4 + TEM (CD44 + CD62L −) cells among T cells in the lung (56.2% [52.2–61] vs. 68% [61.5–78.5]; *p* = 0.0064).

Conversely, the frequencies of CD4 + TCM and CD4 + T naive cells were significantly increased in ATO-treated mice, respectively, by two-fold (4% [3.1–5.1] vs. 1.97% [1.36–3]; *p* = 0.0062 and 5.26% [4.3–12.5] vs. 2.7% [2.22–5.67]; *p* = 0.047) (Fig. 4C). Regarding the CD8 + cell population, treatment with ATO significantly reduced the frequency of CD8 + TEM cells by 31% (47.1% [32.4–56.9] in treated mice vs. 68% in control mice [61.5–78.5]; *p* = 0.0013). Additionally, CD8 + T naive cells were significantly increased by 51% (3.9% [3.4–8.5] vs. 1.94% [1.22–3.26]; *p* < 0.001). However, no differences between groups were observed in the proportion of CD8 + TCM in the lung (Fig. 4D).

### ATO treatment reduces T-cell activation in lung tissue: evidence from RNA-seq analysis

**Fig. 5:**
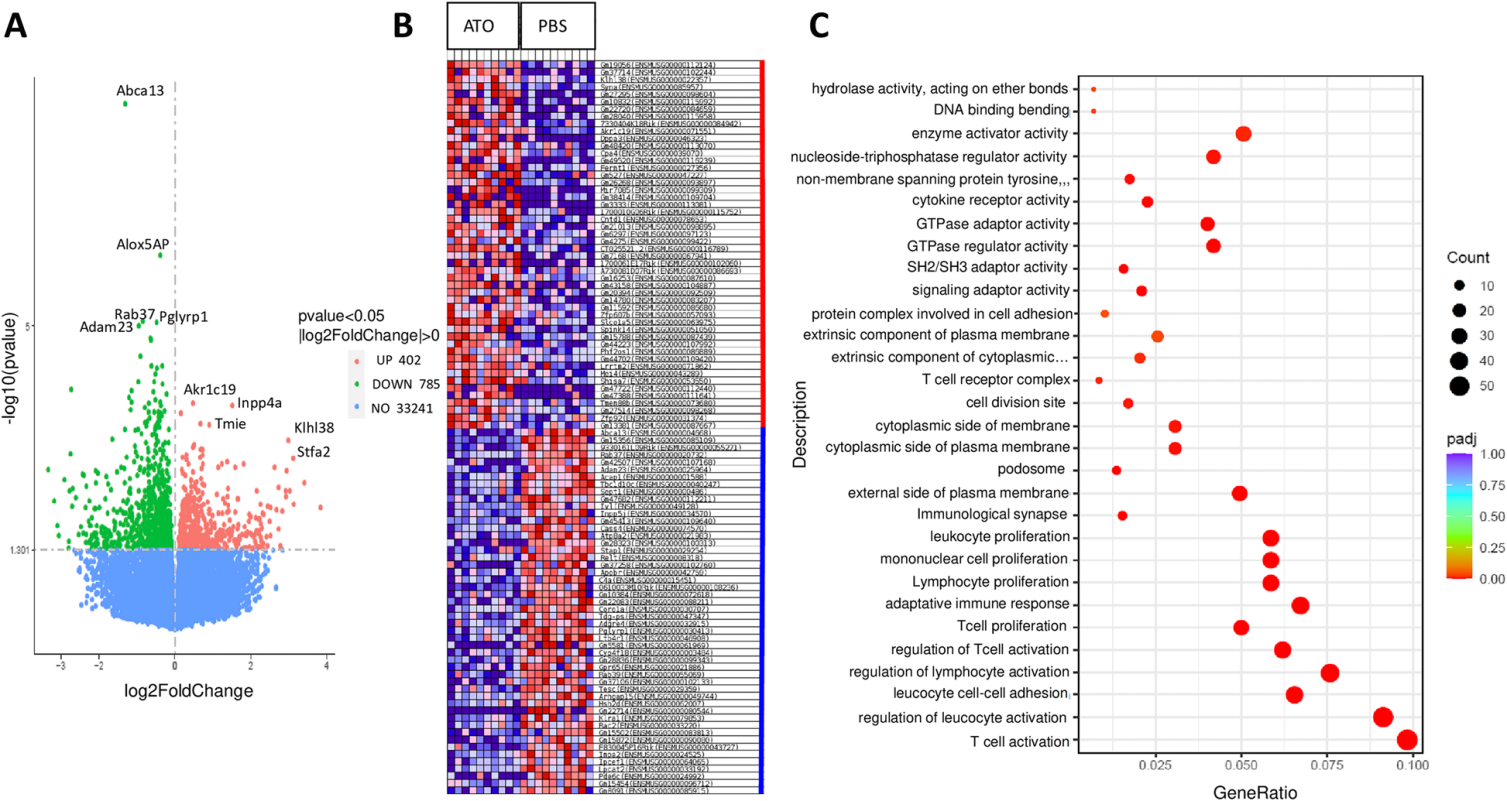
Lung RNA-seq data analysis. **A** Volcano plot representation of differential expression analysis of genes from ATO-treated lungs vs. PBS-treated lungs. Green and red dots mark the genes with significantly decreased and increased expression, respectively. The x-axis shows log2 fold-changes in expression and the y-axis the *p*-value (log10) of a gene being differentially expressed. Corrected *p*-value of 0.05 and absolute foldchange of 2 were set as the threshold. **B** Top 100 differentially expressed genes. The heatmap illustrates the 50 most highly upregulated genes (red) and downregulated genes (blue) in lungs from ATO-treated group (*n* = 10) (left) and PBS group (*n* = 10) (right). **C** GO analysis of genes downregulated in lungs for biological process. *Y*-axis label represents the pathway, and *X*-axis label represents the gene ratio (gene ratio = gene numbers annotated in this pathway term/all gene numbers annotated in this pathway term). The size of the bubble represents the number of genes enriched in the GO terms, and the color shows the padj value of GO terms

Two samples (one from each group) that did not pass quality control tests were excluded for bulk RNA-seq analysis. A total of 1277 differentially expressed genes, including 492 upregulated genes and 785 downregulated genes, were identified in the lungs of ATO-treated mice compared to those treated with PBS (Fig. 5A). The top 5 downregulated genes with *p* value < 1.10^−5^, i.e., -log10(*p* value) > 5 in the ATO group, were *Abca13* (having a role in the innate immune system), *Alox5ap* (an immune-modulating lipid mediator involved in immune response and regulation), *Rab37* (metastasis suppressor involved in lung cancer)*, Pglyrp1* (involved in humoral immune respons*e*), and *Adam23* (a metalloprotease involved in cell adhesion) (Fig. 5A). The top 5 upregulated genes, with *p* value < 0.0005, i.e., -log10(*p* value) > 3, were mostly involved in metabolic processes: *Akr1c19* (oxydoreductase activity), *Inpp4a* (hydrolase activity), *Tmie* and *Stfa2* (involved in protein trafficking), and *Klhl38* (involved in NSCLC via the Akt signaling pathway).

Hierarchical clustering analysis showed that all samples clustered by groups, indicating that lungs from ATO-treated and PBS-treated groups had distinct gene expression profiles (Fig. 5B). The Gene Ontology (GO) enrichment analysis revealed that the significantly (padj < 0.05) downregulated genes expressed in ATO-treated lungs were mostly involved in GO terms for biological process (BP) with 219 GO, molecular function (MF) with 11 GO, and cellular components (CC) with 12 GO. The majority of downregulated biological process pathways in the lungs of ATO-treated mice were associated with immune activity and inflammation, mostly T-cell activation, but also regulation of leucocyte cell–cell adhesion or regulation of general lymphocyte activation (Fig. 5C).

## Discussion

We demonstrated the potential benefits of ATO treatment in Fra2^TG^ mice, a relevant animal model for SSc that recapitulates the severe lung features observed in SSc patients [22]. The findings from this study showed that ATO treatment reduced interstitial lung damage, as measured by the Ashcroft histological scores. Although some measurements did not reach statistical significance, our results suggest that ATO has a favorable effect on lung inflammation. The reduction in macrophages was lower than the one observed for T cells. One limitation is that we did not explore macrophage subsets. However, RNA seq results did not show a difference regarding macrophage differentiation*.*

The immunological effects of ATO observed in the herein study are the following. With a focus on the measurements in the lung which is our primary target, we found an effect at the cellular level with a shift toward a reduced number of effector memory T cells mirroring an increase in naïve T cells. We assume that reduced inflammation, cytokines in the milieu, and Ag exposure may explain such findings. In addition, lung T cells exhibited a reduced activation supported by lower CD69 and PD-1 expression. Finally, we explored gene expression with bulk RNA seq that definitely showed a shift in T cells response, activation, and immune interaction on lung cellular extracts, in line with the known ATO effect.

ATO treatment had clear effects on immune cells and inflammation, but its effects on fibrosis were less pronounced. This is consistent with previous studies showing ATO’s inhibitory effect on T lymphocyte activation in animal models of diabetes and islet allotransplantation [23]. The most striking effects were observed on the pulmonary vascular disease. Indeed, we found a reduction of vascular remodeling demonstrated by a significant decrease in both medial wall thickness and muscularized distal pulmonary arteries, in agreement with a reduction of right ventricular systolic pressure.

Future studies may investigate the possibility of earlier intervention, increasing the dose or combining ATO with other treatments to optimize its effects. Recent studies have shown that the use of divalent metallic cations, such as Cu^2+^, can potentiate ATO’s effects on immune cells and decrease the expression of early markers of fibrosis in animal models of SSc [24, 25]. These findings suggest that ATO may have potential therapeutic applications in the treatment of SSc-related lung diseases.

Overall, the results presented in this study are very promising with regard to interstitial lung disease and pulmonary hypertension, which are believed to be the leading cause of mortality in human disease. The pathophysiological origins of these pathogenic processes are not clear, but the similarities between the Fra2^TG^ animal models and human disease make any improvement in the Fra2 mice very interesting. These observations suggest that ATO could be a promising candidate for the treatment of interstitial lung disease and pulmonary hypertension in human disease. Further studies are needed to explore its potential as a therapeutic option in humans. In oncological diseases, ATO has been shown to inhibit hedgehog signaling via blocking the transcription factor Gli2 [26]. Hedgehog signaling and accumulation of Gli1 and Gli2 have been reported to have a role in scleroderma [27]. In the herein experiment, using RNAseq data, we could not identify any influence of ATO on Gli2.

In a phase II study involving humans with moderate to severe chronic GVH disease, ATO exhibited significant positive effects [28]. The study included 21 patients who participated in a prospective national multicenter single-arm open-label phase II study. The overall response rate at 6 months was 75.0% (95% CI, 50.9 to 91.3%), with 82.4% in the per-protocol patient population. The progression-free survival rate was 95.0% (95% CI, 69.5 to 99.3%) at 6 months and 84% (95% CI, 57.7 to 94.5%) at 12 months. The mean corticosteroid dose generally hugely decreased as early as 6 weeks after the start of the treatment. Therefore, the combination of ATO and steroids was found to be an effective first-line treatment for cGVHD after previous allo-HSCT, with a high clinical response rate and rapid steroid sparing [28].

A phase IIa, open-label, dose-escalating study had previously enrolled 11 adult patients with active systemic lupus erythematosus (SLE) to investigate the efficacy and safety of ATO [29]. The safety profile was good, with four serious adverse events, only two of which were attributable to ATO (neutropenia in 2 patients cotreated with mycophenolate). At week 24, five out of 10 patients were SLE Responder Index 4 (SRI-4) responders. The mean corticosteroid dosage decreased from 11.25 mg/day at baseline to 6 mg/day at week 24. In the long term, six patients achieved low-disease activity criteria [29].

A potential limitation of our study is that we investigated the effects of ATO in a single pre-clinical model. However, our findings are consistent with previous studies that demonstrated the benefits of ATO treatment in autoimmune animal models, including hypochloride-induced sclerodermatous skin disease and sclerodermatous GVHD mouse models. Our results support the early use of ATO in SSc patients due to its predominant anti-inflammatory effects and vessel anti-remodeling effects. In the future, it would be interesting to explore the use of combination therapy with other drugs that can target various aspects of the pathophysiology of the disease. Indeed, we suggest that the next step should be to investigate the potential of combining ATO with targeted anti-fibrotic therapies.

## Conclusion

Our study provides compelling evidence of the positive effects of ATO treatment on lung function in a mouse model of SSc. These effects were characterized by a significant reduction in inflammatory infiltration and strong improvements in vascular remodeling, although the impact on fibrotic features was incomplete. We demonstrate that these benefits are mediated through positive immune improvements, particularly T-cell differentiation and activation. Our findings represent a substantial advancement in understanding the complex interplay between inflammation-driven fibrosis and the pathophysiology of SSc. The clinical translation of our results to patients will require further investigation in future follow-up studies. Our results also pave the way for potential innovative therapies, especially in the early/inflammatory phase of SSc.

### Supplementary Information

**Additional file 1.**

**Additional file 2.**

**Additional file 3.**

## Data Availability

The datasets used and/or analyzed during the current study are available from the corresponding author on reasonable request. All data generated or analyzed for this study are included in this published article.

## Abbreviations

AF: AlexaFluor
α-SMA: Alpha-smooth Muscular actin
AP-1: Activator protein 1
APL: Acute promyelocytic leukemia
ATO: Arsenic trioxide
BSA: Bovine serum albumin
CD: Cluster differentiation
CTLA-4: Cytotoxique-T-lymphocyte-antigen 4 protein
DAB: Diaminobenzidine
FBS: Fetal bovine serum
Fra-2: Fos-related antigen
FRC: Functional residual capacity
GO: Gene ontology
GVHD: Graft-versus-host disease
HE: Hematoxylin-eosin
HLA: Human leucocyte antigen
HRP: Horseradish peroxidase
HOCL: Hypochlorous acid
IHC: Immunohistochemistry
ICOS: Inducible T cell costimulatory
ILD: Interstitial lung disease
micro-CT: Micro-computed tomography
NSCLC: Non-small-cell lung carcinoma
OX40L: Tumor necrosis factor ligand superfamily member 4
PBS: Phosphate buffer saline
PD-1: Programmed cell death
pDC: Plasmacytoide dendritic cells
PH: Pulmonary hypertension
RVSP: Right ventricular systolic pressure
SHG: Second harmony generation
SLE: Systemic lupus erythematosus
SPF: Specific pathogen free
SSc: Systemic sclerosis
TCM: T central memory
TEM: T effector memory
TG: Transgenic
TPEF: Two-photon excited fluorescence

## References

[1] Elhai M, Avouac J, Kahan A, Allanore Y (2015). Systemic sclerosis: recent insights. Joint Bone Spine mai.

[2] Elhai M, Meune C, Avouac J, Kahan A, Allanore Y (2012). Trends in mortality in patients with systemic sclerosis over 40 years: a systematic review and meta-analysis of cohort studies. Rheumatology (Oxford).

[3] Varga J, Trojanowska M, Kuwana M (2017). Pathogenesis of systemic sclerosis: recent insights of molecular and cellular mechanisms and therapeutic opportunities. J Scleroderma Relat Disord.

[4] Brown M, O’Reilly S (2018). The immunopathogenesis of fibrosis in systemic sclerosis. Clin Exp Immunol.

[5] Orvain C, Assassi S, Avouac J, Allanore Y (2020). Systemic sclerosis pathogenesis: contribution of recent advances in genetics. Curr Opin Rheumatol.

[6] Truchetet ME, Brembilla NC, Chizzolini C. Current concepts on the pathogenesis of systemic sclerosis. Clin Rev Allergy Immunol. 2023;64(3):262–83. 10.1007/s12016-021-08889-8 PMC10167130 34487318

[7] Avouac J, Elhai M, Allanore Y (2013). Experimental models of dermal fibrosis and systemic sclerosis. Joint Bone Spine..

[8] Maurer B, Distler JHW, Distler O (2013). The Fra-2 transgenic mouse model of systemic sclerosis. Vascul Pharmacol mars.

[9] Maurer B, Reich N, Juengel A, Kriegsmann J, Gay RE, Schett G (2012). Fra-2 transgenic mice as a novel model of pulmonary hypertension associated with systemic sclerosis. Ann Rheum Dis août.

[10] Elhai M, Avouac J, Hoffmann-Vold AM, Ruzehaji N, Amiar O, Ruiz B (2016). OX40L blockade protects against inflammation-driven fibrosis. Proc Natl Acad Sci U S A.

[11] Boleto G, Guignabert C, Pezet S, Cauvet A, Sadoine J, Tu L (2018). T-cell costimulation blockade is effective in experimental digestive and lung tissue fibrosis. Arthritis Res Ther.

[12] Orvain C, Cauvet A, Prudent A, Guignabert C, Thuillet R, Ottaviani M (2022). Acazicolcept (ALPN-101), a dual ICOS/CD28 antagonist, demonstrates efficacy in systemic sclerosis preclinical mouse models. Arthritis Res Ther.

[13] Douer D, Hu W, Giralt S, Lill M, DiPersio J (2003). Arsenic trioxide (trisenox) therapy for acute promyelocytic leukemia in the setting of hematopoietic stem cell transplantation. Oncologist.

[14] Gao C, Jiang J, Ma P, Cheng P, Lian Y, Zhao B (2015). Arsenic trioxide induces T cell apoptosis and prolongs islet allograft survival in mice. Transplantation sept.

[15] Kavian N, Marut W, Servettaz A, Laude H, Nicco C, Chéreau C (2012). Arsenic trioxide prevents murine sclerodermatous graft-versus-host disease. J Immunol..

[16] Kavian N, Marut W, Servettaz A, Nicco C, Chéreau C, Lemaréchal H (2012). Reactive oxygen species-mediated killing of activated fibroblasts by arsenic trioxide ameliorates fibrosis in a murine model of systemic sclerosis. Arthritis Rheum.

[17] Birnhuber A, Biasin V, Schnoegl D, Marsh LM, Kwapiszewska G (2019). Transcription factor Fra-2 and its emerging role in matrix deposition, proliferation and inflammation in chronic lung diseases. Cell Signal déc.

[18] Ashcroft T, Simpson JM, Timbrell V (1988). Simple method of estimating severity of pulmonary fibrosis on a numerical scale. J Clin Pathol avr.

[19] Ponsoye M, Frantz C, Ruzehaji N, Nicco C, Elhai M, Ruiz B (2016). Treatment with abatacept prevents experimental dermal fibrosis and induces regression of established inflammation-driven fibrosis. Ann Rheum Dis déc.

[20] Avouac J, Konstantinova I, Guignabert C, Pezet S, Sadoine J, Guilbert T (2017). Pan-PPAR agonist IVA337 is effective in experimental lung fibrosis and pulmonary hypertension. Ann Rheum Dis.

[21] Chaumais MC, Djessas MRA, Thuillet R, Cumont A, Tu L, Hebert G (2021). Additive protective effects of sacubitril/valsartan and bosentan on vascular remodelling in experimental pulmonary hypertension. Cardiovasc Res..

[22] Allanore Y, Simms R, Distler O, Trojanowska M, Pope J, Denton CP (2015). Systemic sclerosis. Nat Rev Dis Primers.

[23] Zhao B, Xia JJ, Wang LM, Gao C, Li JL, Liu JY (2018). Immunosuppressive effect of arsenic trioxide on islet xenotransplantation prolongs xenograft survival in mice. Cell Death Dis.

[24] Chêne C, Jeljeli MM, Rongvaux-Gaïda D, Thomas M, Rieger F, Batteux F (2022). A Fenton-like cation can improve arsenic trioxide treatment of sclerodermatous chronic Graft-versus-Host Disease in mice. Front Immunol.

[25] Chêne C, Rongvaux-Gaïda D, Thomas M, Rieger F, Nicco C, Batteux F (2023). Optimal combination of arsenic trioxide and copper ions to prevent autoimmunity in a murine HOCl-induced model of systemic sclerosis. Front Immunol.

[26] Nakamura S, Nagano S, Nagao H, Ishidou Y, Yokouchi M, Abematsu M (2013). Arsenic trioxide prevents osteosarcoma growth by inhibition of GLI transcription via DNA damage accumulation. PLoS ONE.

[27] Zerr P, Palumbo-Zerr K, Distler A, Tomcik M, Vollath S, Munoz LE (2012). Inhibition of hedgehog signaling for the treatment of murine sclerodermatous chronic graft-versus-host disease. Blood..

[28] Rongvaux-Gaïda D, Dupuis M, Poupon J, Djebrani-Oussedik N, Lemonnier C, Rieger F (2022). High response rate and corticosteroid sparing with arsenic trioxide-based first-line therapy in chronic graft-versus-host disease after allogeneic hematopoietic stem cell Transplantation. Transplant Cell Ther.

[29] Hamidou M, Néel A, Poupon J, Amoura Z, Ebbo M, Sibilia J (2021). Safety and efficacy of low-dose intravenous arsenic trioxide in systemic lupus erythematosus: an open-label phase IIa trial (Lupsenic). Arthritis Res Ther..

