# Supplementary figures and images for: Arsenic trioxide demonstrates efficacy in a mouse model of preclinical systemic sclerosis
Source: Arthritis Res Ther. 2023 Sep 12;25:167. doi: 10.1186/s13075-023-03143-2 (PMC10496169; doi:10.1186/s13075-023-03143-2)

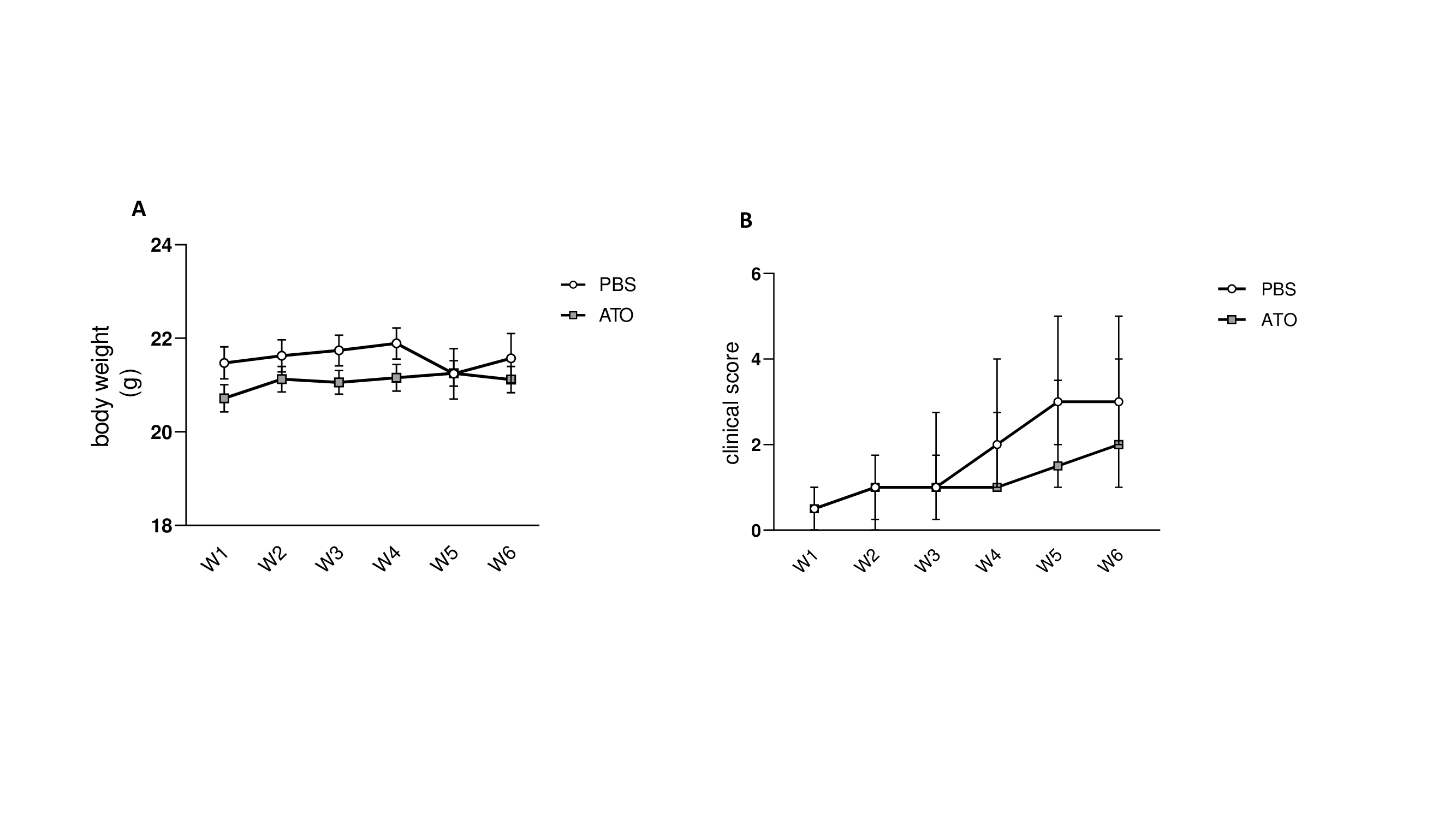

Supplement: Supplementary file 1 — Additional file 1. [file 13075_2023_3143_MOESM1_ESM.tiff]

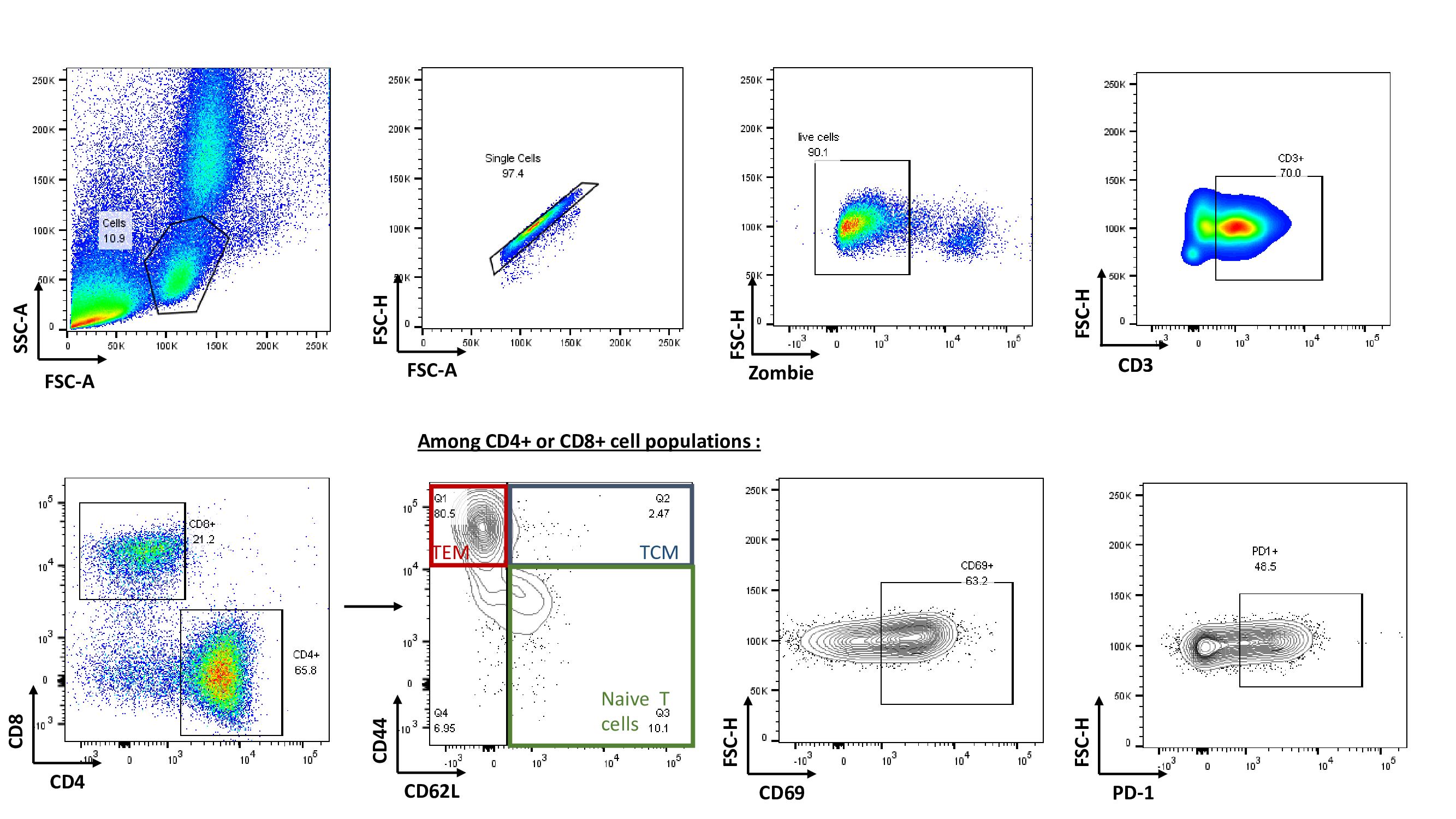

Supplement: Supplementary file 3 — Additional file 3. [file 13075_2023_3143_MOESM3_ESM.tiff]
